# Purification, Characterization, and Oil-Displacement Performance of Rhamnolipid Biosurfactant Produced by *Bacillus* sp. DQ-4

**DOI:** 10.4014/jmb.2601.01038

**Published:** 2026-07-09

**Authors:** Jing Wei, Xiaojun Sun, Liping Pang, Xin Shao, Di Yang, Hongrui Fu, Jinren Lu, Mutai Bao

**Affiliations:** 1 Frontiers Science Center for Deep Ocean Multispheres and Earth System, and Key Laboratory of Marine Chemistry Theory and Technology, Ministry of Education, Ocean University of China, Qingdao 266100, P. R. China; 2 College of Chemistry and Chemical Engineering, Ocean University of China, Qingdao 266100, P. R. China

**Keywords:** Rhamnolipid, Biosurfactant, *Bacillus*, Enhanced oil recovery (EOR), Oil displacement

## Abstract

Rhamnolipids are attractive biosurfactants for enhanced oil recovery, but the commonly used producing strains may raise biosafety concerns. In this study, a rhamnolipid-producing isolate, designated *Bacillus* sp. DQ-4, was obtained from oily sludge and cultivated in a glucose-based fermentation medium. The purified product, DQ-Rha, was characterized by TLC, FTIR, MALDI-TOF MS, and 1D/2D NMR (^1^H, ^13^C, ^1^H-^1^H COSY, ^1^H-^13^C HSQC, and HMBC). The combined spectroscopic results were consistent with a rhamnolipid structure, and no obvious conflicting signals were detected. DQ-Rha reduced the surface tension to 33.4 mN/m, and the DQ-4 supernatant showed an oil-spreading diameter of 89.3 mm and an emulsification index of 72.3% against diesel. In oil-displacement-related evaluations, DQ-Rha gave an oil-washing efficiency of 54.62%. In etched micromodel experiments, the total recovery factor reached 60.06%, which was comparable to that of commercial rhamnolipid (59.53%). In core flooding experiments at 85°C, injection of DQ-Rha after primary water flooding further increased the recovery factor by 10.56%, again showing performance comparable to commercial rhamnolipid. Acute oral toxicity testing in ICR mice showed no mortality or obvious toxic symptoms at 5040.6 mg/kg, and the acute oral LD_50_ was greater than 5000 mg/kg under the test conditions. These results suggested that DQ-Rha was a rhamnolipid biosurfactant with favorable oil-displacement-related performance and low acute oral toxicity.

## Introduction

With the increasing difficulty of global oil exploitation, the limitations of traditional chemical oil-displacing agents, such as synthetic surfactants and polymers, have become increasingly evident [1, 2]. Although these agents can temporarily enhance crude oil recovery [3, 4], their high cost, environmental toxicity (*e.g.*, groundwater contamination caused by alkylbenzene sulfonates) [5-7], and risks of reservoir damage (*e.g.*, pore blockage induced by polymers) severely restrict their sustainable application [8, 9]. Against this backdrop, biosurfactants have emerged as a promising alternative owing to their eco-friendly and biodegradable properties [10-12]. As a representative glycolipid biosurfactant [13-15], rhamnolipid demonstrates remarkable potential in oil recovery by significantly reducing oil-water interfacial tension [16, 17], facilitating oil-water separation [18, 19], and offering environmental compatibility [20-22]. Moreover, its natural origin avoids the reservoir contamination typically associated with chemical agents, aligning well with the requirements of sustainable development [23, 24].

Traditional rhamnolipid production approaches, such as chemical synthesis or plant extraction, are costly, inefficient, and potentially polluting, which limits their large-scale application [13, 25, 26]. With advances in biotechnology, microbial fermentation has become the mainstream method for rhamnolipid production [27-29]. Rhamnolipids are naturally produced by *Pseudomonas aeruginosa* and some *Burkholderia*. Some strains of different bacterial species, such as *P. aeruginosa* NRRL B-30761, have acquired the ability to produce rhamnolipids through horizontal gene transfer [30]. Although some *Burkholderia* strains have been reported as rhamnolipid producers, their reported yields remain relatively low. Arvin *et al*. optimized the conditions of this bacterium, and its yield was 1.66 g/L [31]. Among the producing strains, *P. aeruginosa* has attracted significant attention due to its high biosynthetic efficiency [32-34]. However, this bacterium also presents notable drawbacks. *P. aeruginosa* is an opportunistic pathogen with strong virulence, capable of causing pneumonia [35, 36], urinary tract infections, and other diseases in immunocompromised individuals [37-39]. It also exhibits pronounced antibiotic resistance through mechanisms such as efflux pumps [40, 41], β-lactamase secretion, and biofilm formation, posing challenges for pharmaceutical control during production [42-44]. In addition, its high adaptability enables survival under extreme environmental conditions [45-47]. While this feature may be advantageous in some contexts, during fermentation it can lead to competition with other microorganisms [48, 49], resulting in contamination [50-52]. Therefore, screening for more stable and biosafe microbial strains has become a key strategy for rhamnolipid production.

From the oily sludge of the Oilfield, a rhamnolipid-producing strain, *Bacillus* sp. DQ-4, was isolated. The strain was identified and analyzed by means of morphological observation, physiological and biochemical tests, as well as 16S rDNA gene sequencing. Shake-flask culture experiments were carried out, with the surface tension, oil-spreading diameter and emulsification index of the fermentation broth used as evaluation indicators. The biosurfactant produced (DQ-Rha) was qualitatively analyzed and characterized for its physicochemical properties through Thin-layer chromatography (TLC), Fourier-transform infrared spectroscopy (FTIR), Matrix-assisted laser desorption/ionization time-of-flight mass spectrometry (MALDI-TOF-MS) and Nuclear magnetic resonance spectroscopy (NMR). Furthermore, its effectiveness in enhancing oil recovery was systematically evaluated.

## Materials and Methods

### Chemical Reagents, and Medium Strain

All chemical reagents used in this study were of analytical grade. The oil sludge was obtained from Daqing Oilfield, crude oil (Y102) was provided by the Shengli Oilfield (Density 25°C 0.9416 g/cm^3^, 60°C 0.9189 g/cm^3^, 85°C 0.9021 g/cm^3^).

Inorganic salt culture medium: NaCl 5.0 g/L, K_2_HPO_4_ 3.48 g/L, Na_2_HPO_4_ 1.5 g/L, (NH_4_)_2_SO_4_ 3.96 g/L, MgSO_4_ 0.7 g/L, trace element liquid 1 mL/L, pH 7.0-7.2.

LB medium: peptone 10 g/L, yeast extract 5 g/L, NaCl 5 g/L, pH 7.0-7.2.

Fermentation medium: Glucose 20 g/L, NH_4_Cl 2 g/L, CH_3_COONa 5 g/L, MgSO_4_ 0.2 g/L, MnSO_4_ 0.05 g/L, K_2_HPO_4_ 2 g/L, Beef extract 1.2 g/L, Yeast extract 4 g/L, Peptone 8 g/L, pH 7.0-7.2.

In this study, oil sludge samples were added to the pre-sterilized inorganic salt culture medium to fully release the bacteria in the oil sludge. Then, 5 mL of the resulting bacterial suspension was transferred to LB medium for further enrichment. After isolation and purification, strain DQ-4 was inoculated into the fermentation medium for biosurfactant production. In a 250 mL of conical flask, 100 mL of liquid medium was added and the flask was placed on a rotary shaker at 150 rpm for 72 h of fermentation. The fermentation temperature and initial pH were 15°C and 7, respectively.

Strain DQ-4 was identified by 16S rDNA gene amplification following the procedures described in Bergey’s Manual of Determinative Bacteriology. Sequences with relatively high similarity were selected based on BLAST results, and a phylogenetic tree was constructed in MEGA using the neighbor-joining method (Fig. S2 in the Supporting Information). The 16S rDNA gene sequence of strain DQ-4 showed 100% similarity to that of *Bacillus altitudinis* TM2 (accession no. AP025262.1). Based on the phylogenetic analysis and sequence comparison, the strain was conservatively designated *Bacillus* sp. DQ-4, and its GenBank accession number was PX491225.1. The strain was then deposited in the China General Microbiological Culture Collection Center (CGMCC) under the accession number CGMCC No. 33396.

### Determination of the Activity of Biosurfactants

The fermentation broth was centrifuged at 12,000 rpm for 10 min, and the supernatant was collected. The biosurfactant activity of the *Bacillus* sp. DQ-4 supernatant was determined using oil spreading, surface tension, and emulsification methods. All experiments were conducted in triplicate.

**Oil spreading assay.** The oil spreading assay was used to evaluate surfactant activity by measuring the diameter of the clear zone formed after the surfactant solution was added to the oil-water interface. Briefly, 20 mL of distilled water was added to a 9-cm-diameter Petri dish, followed by 0.5 mL of Sudan Red III-stained n-hexadecane. After the hexadecane had spread evenly over the water surface, 0.02 mL of DQ-4 supernatant was added to the center of the oil film. The diameter of the clear zone was then measured. The experiment was conducted at room temperature.

**Surface tension method.** The surface tension of the culture was measured using the Du-Nouy method with a BZY-2 surface tension meter (Shanghai Hengping Instrument Factory) [53]. Before measurement, the platinum ring was thoroughly cleaned and dried, then immersed in the sample surface, and the surface tension value was recorded once the reading stabilized.

**Emulsification method.** The determination of emulsifying activity was performed according to the method of Cooper and Goldenberg (1987) with slight modifications, assessing the emulsifying activity of *Bacillus* sp. DQ-4 supernatant on diesel fuel. Briefly, 5 mL of diesel fuel was thoroughly shaken with 5 mL of supernatant for 30 s, then left to stand at room temperature for 24 h. The height and stability of the emulsified layer were observed, and the 24 h emulsification index (EI%) was calculated using the following formula:

$$\mathrm{EI}=\frac{h}{H}\times 100\%$$ (1)

Here, *h* and *H* denote the heights of the emulsified layer and the total height, respectively.

### Extraction and Purification of Biosurfactants

After incubation, the fermentation broth was centrifuged at 12,000 rpm for 10 min to remove most of the bacterial cells. The filtrate was adjusted to pH 2.0 using 6 mol/L hydrochloric acid and refrigerated at 4°C for 24 h. An equal volume of ethyl acetate was added for multiple extractions. The organic layers were combined, and the organic solvent was removed by rotary evaporation at 46°C to obtain the crude surfactant product and its mass was accurately weighed.

The crude product was dissolved in anhydrous methanol by ultrasonication. Centrifugation was performed at 12,000 rpm in a refrigerated centrifuge at 4°C for 10 min. The supernatant was evaporated using a rotary evaporator at 46°C to obtain the pure compound (DQ-Rha). The weight of the pure compound DQ-Rha was accurately measured. The extracted pure biosurfactant was subjected to qualitative analysis, acute oral toxicity testing, oil-washing performance evaluation, and core physical simulation oil displacement capacity assessment using the DQ-4 strain.

### Characterization of DQ-Rha

**Preliminary characterization.** The fermentation broth was centrifuged to remove most bacterial cells, and the pH of the resulting supernatant was adjusted to 2.0 using 6 mol/L HCl. The acidified solution was then incubated at 4°C for 24 h. The presence or absence of a white precipitate was recorded and used for the preliminary characterization of the surfactant. When a white precipitate was observed, the surfactant was considered likely to be lipopeptide-or lipoprotein-based. When no white precipitate was observed, the surfactant was considered likely to be glycolipid-based.

**Thin-layer chromatography.** Small amounts of DQ-Rha and commercial rhamnolipid were separately dissolved in methanol. The samples were applied to a silica gel plate along a pencil line drawn 0.5 cm above the bottom edge of the plate. The spot diameter was kept below 2 mm, and each spot was allowed to dry completely before the next application. After the samples had dried completely, the plate was developed in a chamber containing a solvent system of chloroform: methanol: water = 65:25:4 (v/v/v). Development was stopped when the solvent front reached approximately 1 cm below the top edge of the plate. The plate was then visualized with phenol-sulfuric acid reagent. Brown spots were considered consistent with glycolipid components, whereas lipopeptide components remained colorless under these conditions.

**Fourier-transform infrared spectroscopy.** Using Fourier-Transform Infrared Spectroscopy, infrared spectra in the range of 400-4000 cm^-1^ were determined using the KBr pellet method. First, freeze-dried DQ-Rha (1 mg) was mixed with dried KBr (100 mg). Then, the mixture was pressed into a metal mold. The metal mold was then scanned to obtain the infrared absorption spectrum of DQ-Rha for identification and analysis.

**Matrix-assisted laser desorption/ionization time-of-flight mass spectrometry.** The structure of the DQ-Rha biosurfactant was analyzed using matrix-assisted laser desorption/ionization time-of-flight mass spectrometry (MALDI-TOF MS) on an SCIEX 4800 analyzer (Applied Biosystems, USA) with laser intensities ranging from 3200-5500. Using 0.1% (*v/v*) α-cyano-4-hydroxycinnamic acid (CHCA) as the matrix solution, mass measurements were performed in the range of 600-4000 [54-56].

**Nuclear magnetic resonance spectroscopy.** Using a Bruker Avance NEO 500 NMR spectrometer, 20 mg of DQ-Rha was dissolved in deuterated chloroform. The solution was centrifuged at 10,000 rpm for 10 min at room temperature. The NMR spectroscopy of DQ-Rha was performed at 500 MHz using a 5 mm reverse-bladed probe.

### Acute Oral Toxicity Study of the DQ-Rha Biosurfactant

The acute oral toxicity test was conducted by Ningbo Customs Center. The study was performed in accordance with GB/T 21804-2008, Fixed Dose Procedure for Acute Oral Toxicity Testing of Chemicals, and the fixed dose procedure was used to evaluate the acute oral toxicity of DQ-Rha.

**Preparation of test samples.** The samples used in the preliminary test and the main test were prepared in the same way. A measured amount of sample was placed in a beaker. A small volume of sterile water for injection was added and mixed well. The mixture was then transferred to a 20 mL volumetric flask. The beaker was rinsed several times with small volumes of sterile water for injection, and the rinsing solution was combined in the same volumetric flask. The solution was then brought to volume with sterile water for injection, mixed thoroughly, and transferred to a sample tube for later use. All samples were freshly prepared and used immediately. The gavage volume was 0.2 mL/10 g BW.

**Experimental animals.** The experimental animals were SPF-grade ICR mice. A total of five mice were used, and all of them were female, non-pregnant, and nulliparous. Their body weights ranged from 18.5-21.4 g, and the body weight of each animal did not exceed ±20% of the mean body weight for animals of the same sex. The animals were housed in a barrier animal room. The environmental temperature ranged from 21.7 to 22.9°C, and the relative humidity ranged from 39.0% to 48.4%. The animals were provided with primary RO ultrafiltered water. The water was chlorinated, and the free chlorine concentration was maintained at 0.3-2 mg/L. The animals had free access to water through drinking nozzles.

**Dosing procedure and observation.** Before the experiment, the animals were acclimated in the barrier animal room for 6 days. The animals were fasted for 4 h before dosing. After the study began, DQ-Rha was administered once by oral gavage. One animal was first used in the preliminary test, and the actual administered dose was 5040.6 mg/kg. This animal survived for 96 h after dosing. Based on this result, the dose used in the main test was set at 5000 mg/kg. Four additional animals were then used in the main test, and the actual administered dose was 5070.3 mg/kg. After dosing in the main test was completed, no additional animals or dose groups were added, and the study entered the observation period directly. After each administration, the animals were fasted for another 1 h. The observation period lasted 14 days. During this period, signs of toxicity and mortality were recorded, and body weight was measured once a week. At the end of the observation period, all surviving animals were euthanized and subjected to gross necropsy.

### Evaluation of Oil Displacement Performance using Etched Micromodels

The etched glass micromodel oil displacement experiments were conducted at 25°C. Diagonal homogeneous etched glass micromodels with pore radii of approximately 0.2-1 mm were used. Before each experiment, the micromodels were sequentially cleaned with petroleum ether, ethanol, and deionized water and were then rendered hydrophilic. After saturation with formation water, each micromodel was connected to the displacement apparatus, and crude oil Y102 was injected at a constant flow rate of 0.5 μL/min until no further water was produced. The oil-saturated micromodel was then aged for 24 h. After aging, water flooding was performed at the same flow rate until no further oil was produced. Formation water, Rha solution, and DQ-Rha solution were then injected separately at the same flow rate until no further oil was produced. After each injection test, the micromodel was cleaned, and each injection system was tested in duplicate. During the experiments, a high-magnification camera was used to record the oil-water distribution in the micromodel and its dynamic changes during displacement. The residual oil content in the etched glass micromodel was then determined by image analysis, and the oil recovery factor was calculated from the change in residual oil content. The image features were further analyzed to interpret the microscopic oil displacement mechanisms.

### DQ-Rha Oil-Washing Performance Test

Crude oil Y102 was uniformly mixed with quartz sand of different mesh sizes at a mass ratio of 1:5. The mixture was aged at 85°C for 48 h to prepare oil-sand samples. The density of crude oil Y102 used in this experiment was as described in Section 2.1. Approximately 15.0 g of the aged oil-sand mixture was weighed and transferred to an oil-washing bottle. Then, 30 mL of 1% Rha solution or 1% DQ-Rha solution was added separately. The mixtures were shaken at 90 rpm and 85°C for 2 h. Each bottle was then filled to the upper calibration mark with the corresponding solution, mixed thoroughly, and kept at 85°C for another 2 h. The volume of the eluted oil was then recorded. A formation water control was run in parallel. The mass of the aged oil-sand sample and the volume of the eluted oil were recorded, and the oil recovery efficiency was calculated using the following equation:

$$X=\frac{\rho }{k}\left(\frac{V_{1}}{M_{1}}-\frac{V_{2}}{M_{2}}\right)\times 100\%$$ (2)

where *X* represents oil recovery efficiency (%); *V*_1_ is the volume of oil recovered from the experimental group (mL); *V*_2_ is the volume of oil recovered from the control group (mL); *M*_1_ is the oil-sand mass of the experimental group (g); *M*_2_ is the oil-sand mass in the control group (g); *k* is the percentage of oil in the oil-sand (%); *ρ* is the density of crude oil (g/cm^3^).

### Core Physical Simulation Oil Recovery Experiment

Core flooding experiments were conducted using artificial sand-packed cores to evaluate the oil displacement performance of formation water, Rha, and DQ-Rha under reservoir conditions. Three artificial sand-packed cores, designated as #1, #2, and #3, were used in the experiments. Core #1 served as the blank control and received formation water. Core #2 was used to evaluate the commercial rhamnolipid Rha. Core #3 was used to evaluate DQ-Rha, the metabolite produced by strain DQ-4. After cleaning, the cores were placed in a vacuum saturation apparatus and saturated with formation water, and the pore volume (PV) was determined by the gravimetric method. Crude oil Y102 was then injected into the cores at a flow rate of 0.3-0.6 mL/min to displace the formation water in the pore space, and the saturated oil volume was recorded. Both the formation water saturation and crude oil saturation procedures were carried out in accordance with SY/T 6311-2012, Technical Requirements for High-Temperature, High-Pressure Three-Dimensional Scaled Physical Simulation Experiments for Steam Injection Oil Recovery. After oil saturation, the cores were placed in a thermostatic chamber at the preset reservoir temperature and aged for 7 d. After aging, the cores with known parameters were mounted in core holders, and primary water flooding was performed by injecting formation water from the inlet at 1 mL/min for 1 PV. The produced fluids were collected at the outlet, and the recovery factor after primary water flooding was calculated from the cumulative oil production. After primary water flooding, formation water, Rha solution, or DQ-Rha solution was injected into the corresponding core at 1 mL/min for 0.5 PV until the pressure stabilized. Secondary water flooding was then carried out by injecting formation water at 1 mL/min for 1 PV. The produced fluids were collected, and the final recovery factor was calculated from the cumulative oil production during the secondary water-flooding stage. The equations used to calculate PV and recovery factor are given below:

$$v=m_{1}-m_{0}$$ (3)

$$E=\frac{v_{0}}{V}\times 100\%$$ (4)

where *m*_1_ is the weight of the saturated core (g); *m*_0_ is the weight of the dried core (g);

*v* is the pore volume (mL); *V* is the saturated oil volume, i.e., the water production volume (mL); *v*_o_ is the stage oil production volume (mL); *E* is the system recovery rate (%).

## Results and Discussion

### Determination of the Surface Activity of Biosurfactants

**Fig. 1:**
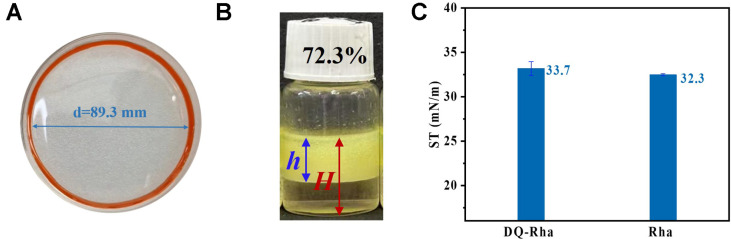
Surface activity analysis and emulsification activity analysis of DQ-4 supernatant. (**A**) Oil spreading, (**B**) Diesel emulsification, (**C**) Surface tension of DQ-Rha and Rha.

The oil displacement activity test can indirectly reflect the strength of the surface activity of the biosurfactants. As shown in Fig. 1A, the supernatant of DQ-4 exhibited a relatively large oil spreading area with a diameter of 89.3 mm in the oil spreading test, indicating that the biosurfactant produced by *Bacillus* sp. DQ-4 has strong surface activity. The *Bacillus* sp. DQ-4 supernatant was vigorously mixed with diesel and left at room temperature for 24 h. Fig. 1B shows the emulsification activity, and the emulsification index was measured at 72.3%. Under greenhouse conditions, the surface activities of DQ-Rha and Rha were compared by measuring surface tension, as shown in Fig. 1C. The surface tension values of DQ-Rha and Rha decreased to 33.4 and 32.3 mN/m, respectively. These results indicate that DQ-4 supernatant has high surface activity and is a potential producer of surface-active molecules.

### Characterization of DQ-Rha

**Fig. 2:**
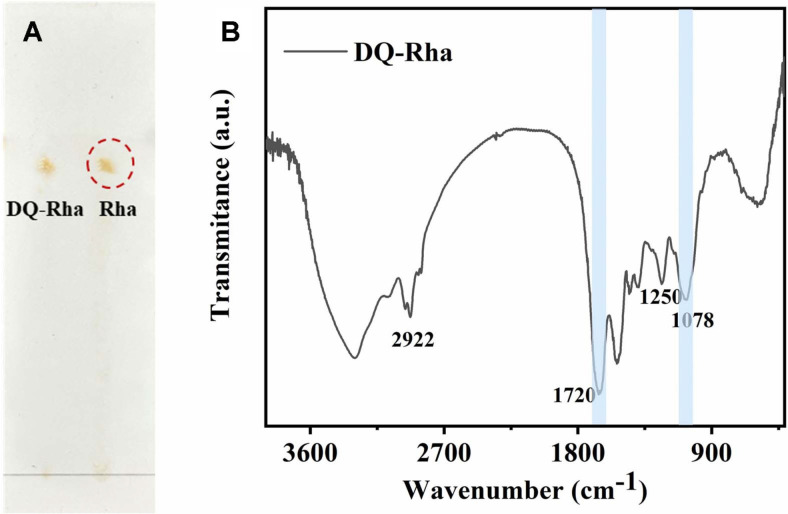
The pictures of TLC analysis (A) and FTIR spectrum (B) of DQ-Rha.

**Thin-layer chromatography and fourier-transform infrared spectroscopy.** Purified DQ-Rha was subjected to thin-layer chromatography. Chloroform/methanol/water was used as the developing agent, and commercial rhamnolipid (Rha) was used as the control. The chromatographic results are shown in Fig. 2A. Brown spots appeared after the plate was sprayed with phenol-sulfuric acid reagent. Compared with the control, the Rf value and the degree of color development were consistent.

FT-IR analysis was used to characterize the functional groups of DQ-Rha, and the results are shown in Fig. 2B. Analysis indicated that the biosurfactant DQ-Rha produced by *Bacillus* sp. DQ-4 exhibited characteristic absorption peaks at 2920 and 2850 cm^-1^ (-CH_2_-/-CH_3_ cm^−1^), corresponding to the stretching vibration of -CH and bending motion of -CH groups, respectively. The characteristic peak at 1720 cm^-1^ indicates the presence of a carbonyl group (-C=O in -COOH). The symmetric stretching vibration of C-O-C at 1000-1100 cm^-1^ confirms the existence of glycosidic bonds and a cyclic lactone structure within the molecule [57, 58]. These findings further confirm that DQ-Rha is a rhamnolipid biosurfactant.

**Fig. 3:**
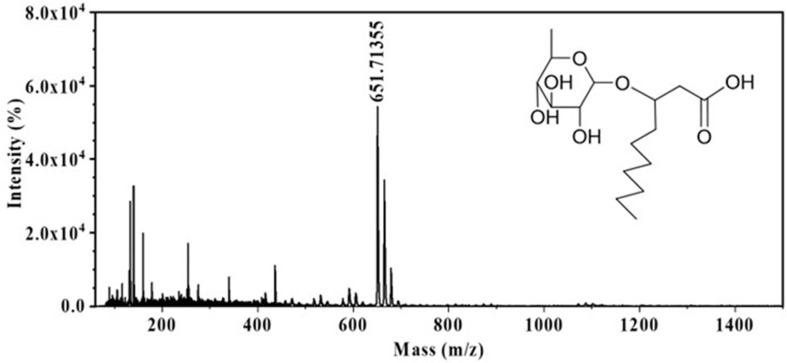
MALDI-TOF MS analysis diagram of DQ-Rha.

**Matrix-assisted laser desorption/ionization time-of-flight mass spectrometry.** Further MALDI-TOF-MS analysis revealed that the observed ion peak was consistent with the presence of a rhamnolipid-type glycolipid, as shown in Fig. 3. Where the mass-to-charge ratio (*m/z*) 651.7 represents a sodium-adduct molecular ion of the rhamnosyl lipid. This peak corresponds to the intact rhamnosyl lipid molecule. The cluster ion consists of one rhamnose unit (molecular weight 146 Da) linked to a long-chain fatty acid (*e.g.*, C10 or C12) [54, 59, 60]. The fragment ion at *m/z* 254 likely represents a cleaved portion of the fatty acid chain from the rhamnosylglycerol, supporting the identification of DQ-Rha as a monomeric rhamnosylglycerol-type biosurfactant.

**Fig. 4:**
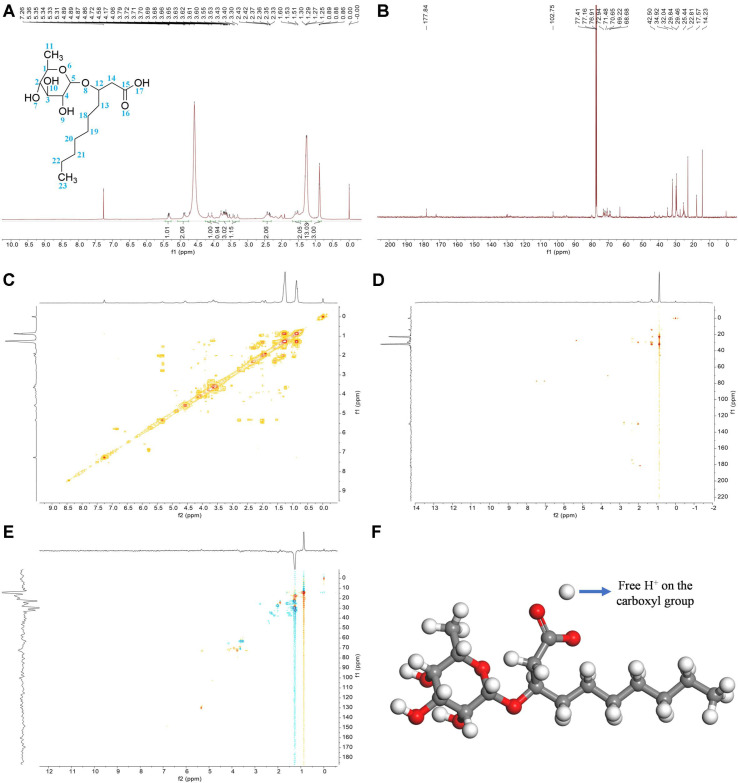
NMR spectra of DQ-Rha (CDCl_3_, 500 MHz): (**A**) ^1^H NMR spectrum; (**B**) ^13^C NMR spectrum; (**C**) ^1^H-^1^H COSY spectrum; (**D**) HMBC spectrum; (**E**) ^1^H-^13^C HSQC spectrum; (**F**) chemical structure of DQ-Rha

**Nuclear magnetic resonance spectroscopy.** To further confirm the molecular structure of DQ-Rha, a systematic analysis was carried out using ^1^H NMR, ^13^C NMR, and two-dimensional NMR spectra, including ^1^H-^1^H COSY, ^1^H-^13^C HSQC, and HMBC. As shown in Fig. 4A, the ^1^H NMR spectrum of DQ-Rha displayed characteristic features of the coexistence of a sugar head group and a fatty acid chain. The multiplet at δ 5.39-5.27 ppm was assigned to the anomeric proton of the rhamnose unit (H-5), and its representative chemical shift was recorded as δ 5.27 ppm. The multiplet at δ 4.96 4.80 ppm was assigned to the hydroxyl protons (H-7, H-9), whereas the singlet at δ 4.06 ppm corresponded to another hydroxyl proton (H-10). The multiplet at δ 4.16 ppm was assigned to the proton on the side-chain carbon linked to an oxygen atom (H-12). Together with the subsequent ^13^C and 2D NMR data, this signal supported the presence of an ether linkage between the sugar ring and the fatty acid chain. The remaining sugarring protons were mainly distributed in the δ 3.86−3.20 ppm region. Among them, the multiplets at δ 3.86−3.53 ppm were assigned to H-1, H-3, and H-4, whereas the multiplets at δ 3.49−3.20 ppm were assigned to H-2, indicating that the rhamnose head group remained intact. In the fatty acid chain region, the multiplets at δ 2.56−2.31 ppm were assigned to the α-methylene protons adjacent to the carboxyl group (H-14), and the multiplets at δ 1.70−1.48 ppm were assigned to the methylene protons adjacent to the oxygenated carbon (H-13). In addition, the broad multiplet at δ 1.40−1.17 ppm mainly corresponded to the methylene protons of the long alkyl chain (H-18, H-19, H-20, H-21, and H-22), but it also included the rhamnose methyl proton (H-11). Although H-11 partially overlapped with the aliphatic methylene envelope, it could still be assigned on the basis of the subsequent 2D NMR data. The triplet at δ 0.88 ppm (t, J = 5.9 Hz) was assigned to the terminal methyl protons of the fatty acid chain (H-23) [61]. Taken together, the ^1^H NMR results indicated that DQ-Rha contained both a rhamnose head group and a fatty acid chain, which was consistent with the rhamnolipid structure shown in Fig. 4F.

The ^13^C NMR spectrum further confirmed the carbon skeleton of DQ-Rha (Fig. 4B). The signal at δ 177.84 ppm was assigned to the carboxyl carbon (C-15), indicating that the terminal carboxyl group of the fatty acid chain was retained in the molecule. The signal at δ 102.75 ppm was assigned to the anomeric carbon of the rhamnose unit (C-5), and this downfield signal was consistent with the typical feature of an anomeric carbon in a sugar ring. The signals from the sugar ring and the oxygen-bonded carbons were mainly distributed in the δ 72.94-68.68 ppm range. Among them, the signal at δ 72.94 ppm was assigned to the side-chain carbon linked to an oxygen atom (C-12), supporting the connection of the fatty acid chain to the sugar ring through an ether bond. The signals at δ 71.48 ppm (C-4), 70.65 ppm (C-3), 69.22 ppm (C-2), and 68.68 ppm (C-1) were assigned to the oxygenated carbons of the rhamnose ring. In the fatty acid chain region, the signal at δ 42.50 ppm was assigned to the α-methylene carbon adjacent to the carboxyl group (C-14), and the signal at δ 34.92 ppm was assigned to the methylene carbon adjacent to the oxygenated carbon (C-13). The long alkyl chain signals appeared at δ 32.04 ppm (C-21), 29.84 ppm (C-20), 29.46 ppm (C-19), 25.44 ppm (C-18), and 22.81 ppm (C-22), which showed the typical carbon signal pattern of a fatty acid chain. Notably, two methyl carbon signals were also observed. The signal at δ 17.57 ppm was assigned to the rhamnose methyl carbon (C-11), which is one of the characteristic features of the rhamnose head group. The signal at δ 14.23 ppm was assigned to the terminal methyl carbon of the fatty acid chain (C-23), reflecting the presence of the long aliphatic tail. The difference in chemical shift between these two methyl carbons indicated that they were located in two distinct chemical environments, namely the sugar head group and the fatty acid tail, which further supported the conclusion that DQ-Rha contained both a rhamnose moiety and a fatty acid chain.

The key connectivity of DQ-Rha was further analyzed by combining the ^1^H-^1^H COSY (Fig. 4C), HMBC (Fig. 4D), and ^1^H-^13^C HSQC (Fig. 4E) spectra. In the ^1^H-^1^H COSY spectrum, the correlation between signals at δ 5.27/3.53 ppm corresponded to the vicinal coupling between H-5 and H-4 in the sugar ring, whereas the correlation between δ 4.16/2.56 ppm corresponded to the vicinal coupling between H-12 and H-14. In addition, the correlation between δ 3.86/1.40 ppm supported coupling between the rhamnose methyl proton (H-11) and the adjacent sugar-ring proton, which further supported the assignment of H-11. The correlation between δ 1.70/1.40 ppm indicated a continuous connection between the proximal methylene group of the side chain and the methylene groups of the long alkyl chain. The correlation between δ 1.17/0.88 ppm further supported the presence of the terminal CH2-CH3 fragment in the fatty acid chain. The HMBC spectrum provided additional long-range evidence for the connectivity of the molecular skeleton. In particular, the correlation at δ C 177.84 ppm/δ H 2.56 ppm indicated connectivity between the carboxyl carbon C-15 and the α-methylene proton H-14, which supported the presence of the terminal carboxyl group in the hydroxy fatty acid chain and further showed that the connectivity of the fatty acid chain fragment was consistent with the proposed structure.

The ^1^H-^13^C HSQC spectrum was mainly used to establish one-to-one correlations between proton and carbon signals. The correlation at δ_C_ 102.75 ppm/δ_H_ 5.27 ppm corresponded to the anomeric carbon C-5 and H-5 of the rhamnose unit, whereas the correlations at δ_C_ 72.94 ppm/δ_H_ 4.16 ppm, δ_C_ 70.65 ppm/δ_H_ 3.86 ppm, and δ_C_ 14.23 ppm/δ_H_ 0.88 ppm corresponded to C-12/H-12, C-3/H-3, and the terminal methyl group C-23/H-23 of the fatty acid chain, respectively. Taken together, the major signals of DQ-Rha and their connectivity were consistent with the rhamnolipid structure shown in Fig. 4F, and no obvious contradictory or abnormal signals were detected. These results further supported the conclusion that the prepared product DQ-Rha was the target rhamnolipid.

### Acute Oral Toxicity Test

Because surfactants used for oil displacement were generally difficult to recover completely after injection into the formation, safety was an important prerequisite for evaluating their application feasibility. The acute oral toxicity test in this study was conducted by the Ningbo Customs Technical Center. The results showed that at a dose of 5040.6 mg/kg, no signs of toxicity or mortality were observed within 14 days following DQ-Rha administration. Body weight gain in the surviving animals remained normal (Table 1), and gross necropsy at the end of the observation period revealed no significant pathological changes. Under the conditions of this study, the acute oral LD_50_ of DQ-Rha in ICR mice was considered to be greater than 5000 mg/kg. These results suggested that DQ-Rha had low acute oral toxicity under the present test conditions. According to toxicological data reported by the U.S. EPA for rhamnolipid biosurfactants, no deaths were observed after a single oral dose of 5000 mg/kg, and the acute oral LD_50_ was greater than 5000 mg/kg, although transient responses such as perianal soiling and diarrhea were reported shortly after administration. In contrast, no obvious poisoning symptoms were observed in the present study. This finding suggested that the acute oral safety of DQ-Rha was generally consistent with that reported for other rhamnolipid systems under comparable test conditions. Overall, these results supported the relatively low acute oral toxicity of the DQ-Rha oil displacement system and indicated that it may have potential for use as an environmentally compatible additive in oilfield applications. Future studies should further evaluate its dermal and inhalation toxicity to establish a more comprehensive safety profile and provide a stronger basis for practical application.

### Evaluation of Oil Displacement Performance Using Etched Micromodels

**Fig. 5:**
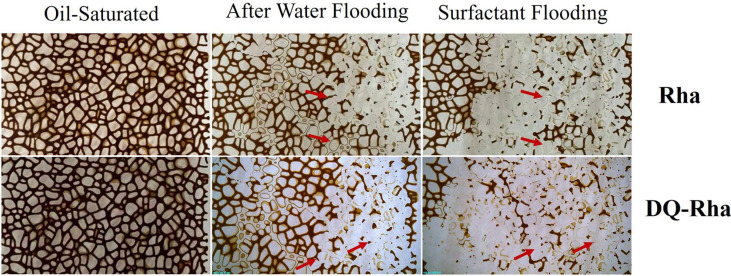
Oil displacement efficiencies of Rha and DQ-Rha after water flooding, and microscopic visualization of the residual oil displacement process.

Microscopic oil recovery tests were conducted using etched micromodels. The oil–water distribution patterns in the models at different displacement stages are shown in Fig. 5. After the first water-flooding cycle, the crude oil content in the model decreased markedly, and a substantial amount of crude oil was displaced from the pore throats. The overall color of the model also became lighter, and the water-flooding recovery factor reached 26.04%. However, after water flooding, a considerable amount of crude oil still remained in the pore space. After the injection of Rha and DQ-Rha, the recovery factors increased to 59.53% and 60.06%, respectively, which were 33.49% and 34.02% higher than that of the water-flooding stage. The micromodel displacement performance of DQ-Rha was therefore comparable to that of commercial Rha. These results suggested that DQ-Rha and commercial Rha showed similar displacement capacity in the micromodel.

During water flooding, the injected water primarily entered larger pore channels, whereas its sweep in smaller pore regions remained limited. Consequently, the residual oil after water flooding mainly occurred as columnar, blind-end, and film-like residual oil, as shown in the figure. After the injection of surfactants, different types of residual oil were mobilized to varying degrees: columnar residual oil gradually deformed and was displaced following a reduction in interfacial tension; dead-end residual oil was passively mobilized due to enhanced lateral sweep; and film-like residual oil was more readily detached as the rock surface became more water-wet. At the same time, some of the residual oil was emulsified into small oil droplets and transported with the aqueous phase. These results suggested that rhamnolipids primarily enhanced oil recovery by reducing the oil-water interfacial tension, altering rock wettability, and promoting the emulsification and dispersion of crude oil.

### Oil-Washing Performance Test

**Fig. 6:**
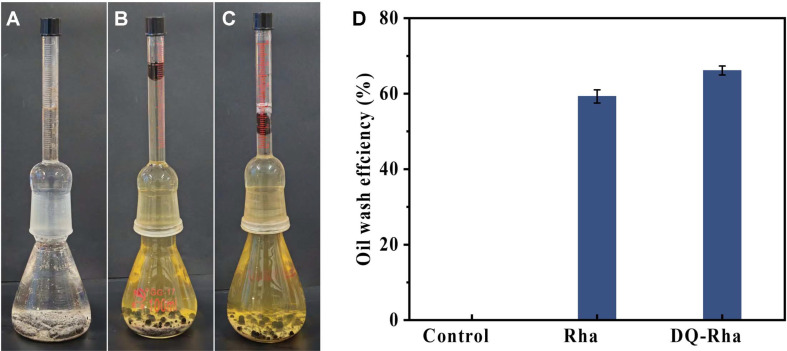
Oil washing performance test: (**A**) Control, (**B**) Rha, (**C**) DQ-Rha, (**D**) Oil washing efficiency.

The oil-stripping performance of Rha and DQ-Rha was evaluated, and the results were shown in Fig. 6. Compared with the blank control, both surfactant systems showed appreciable oil-stripping performance, with efficiencies of 52.14% and 54.62%, respectively. In comparison with previous reports, Zhao *et al*. [62] found that rhamnolipid systems with a higher proportion of mono-rhamnolipids showed improved oil recovery efficiency from oil-in-water emulsions, with a maximum value of 53.81%. Taken together, these results suggested that DQ-Rha may be a promising candidate for MEOR applications and provided a basis for further field evaluation.

### Core Physical Simulation Oil Recovery Experiment

**Fig. 7:**
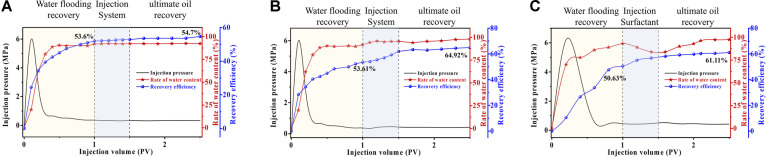
Physical simulation core oil displacement experiments: (**A**) Formation water, (**B**) Rha, (**C**) DQ-Rha.

Dynamic core flooding evaluations of the oil displacement systems constructed with Rha and DQ-Rha were conducted at 85°C using a physical oil displacement apparatus, and the results are shown in Fig. 7. Formation water served as the blank control group. When the cumulative injection volume reached 2.5 PV, the recovery rate increased by only 1.10%. The commercial rhamnolipid (Rha) system achieved a recovery rate of 53.61% during the primary water flooding stage. After injection of the Rha system, the recovery rate further increased by 11.31% compared with primary water flooding alone. The DQ-Rha system achieved a recovery rate of 50.63% during the primary water flooding stage. After injection of the DQ-Rha system, the recovery rate further increased by 10.56% compared with primary water flooding alone. The oil recovery performance of DQ-Rha was comparable to that of the commercial Rha system, which suggested that the two systems had similar oil recovery capacities. These results provided a basis for the further development of efficient oil displacement systems and suggested that DQ-Rha may have potential for MEOR applications.

## Conclusion

In summary, *Bacillus* sp. DQ-4 was identified as a rhamnolipid-producing isolate, and its biosurfactant product, DQ-Rha, was purified and characterized. The combined TLC, FTIR, MALDI-TOF MS, and 1D/2D NMR data supported the identification of DQ-Rha as a rhamnolipid. DQ-Rha showed favorable physicochemical and oil-displacement-related properties, with a yield of 14.263 g/L, a surface tension of 33.4 mN/m, and an oil-washing efficiency of 54.62%. In core flooding experiments, DQ-Rha further increased oil recovery by 10.56% after primary water flooding, showing performance comparable to commercial rhamnolipid. Acute oral toxicity testing indicated that the acute oral LD_50_ of DQ-Rha in ICR mice was greater than 5000 mg/kg under the tested conditions. These results suggested that DQ-Rha represents a green and sustainable additive for oilfields application, with potential for broader use.

## Supplemental Materials

Supplementary data for this paper are available on-line only at http://jmb.or.kr.



## Figures and Tables

**Table 1:** Results of the acute oral toxicity test of DQ-Rha biosurfactant.

Group	Gender	Number of animals (n)	Body weight ($\bar{X}$±SD) (g)				Number of deaths (n)	Mortality rate (%)
			0 days	7 days	14 days	Gain weight in 14 days		
Preliminary test	Female	1	18.9	23.6	27.8	8.9	0	0
Main test	Female	4	20.1±1.40	25.1±1.45	29.8±1.16	9.7±0.92	0	0
