# Supplementary material for: Purification, Characterization, and Oil-Displacement Performance of Rhamnolipid Biosurfactant Produced by Bacillus sp. DQ-4
Source: J Microbiol Biotechnol. 2026 Jul 9;36:e2601038. doi: 10.4014/jmb.2601.01038 (PMC13373720; doi:10.4014/jmb.2601.01038)
Supplement: Supplementary file 1 [file jmb-36-e2601038-supple.pdf]

J. Microbiol. Biotechnol. 2026. 36: e2601038

https://doi.org/10.4014/jmb.2601.01038

Supplementary Figures

Purification, Characterization, and Oil-Displacement Performance of Rhamnolipid Biosurfactant Produced by *Bacillus sp.* DQ-4

Text S1. Determination of the enrichment growth curve of strain DQ-4.

The screened strain DQ-4 was inoculated into LB medium and incubated in a constant-temperature shaker at 30°C and 150 rpm. The microbial growth curve can be divided into four distinct phases: lag, logarithmic, stationary, and decline. The optical density (OD_{600}) of strain DQ-4 was measured at different time intervals to monitor its growth, as shown in Fig. S1. DQ-4 entered the logarithmic phase within 2 h after inoculation, during which the cell population increased exponentially. During this stage, the bacteria exhibited strong adaptability; therefore, inoculating logarithmic-phase cells into fresh medium was found to promote rapid growth and shorten the overall cultivation time. After 24 h, the cells entered the stationary phase, where a large number of secondary metabolites were produced. Strain DQ-4 demonstrated a relatively long stationary phase in this medium, which is favorable for the biosynthesis of secondary metabolites such as biosurfactants. Consequently, a 24 h seed culture was selected for all subsequent experiments.

Text S2. Molecular analysis and identification.

The bacterial was identified by sequencing its 16S rRNA. DNA extraction of strain was using bacterium genomic DNA kit by BGI Co., Ltd. (China) according to the instructions. The 16S universal primer pair 27 F (5′-AGAGTTTGATCCTGGCTCAG-3′) and 1492R (5′-GGTTACCTTGTTACGACTT-3′) were used for PCR amplification.

The reaction system consisted of 0.5 μL genome DNA, 12.5 μL enzyme, 0.6 μL primers, and 11.4 μL dd H_2O. PCR cycle conditions were as follows: 96°C for 5 min, 35 cycles of denaturation at 96°C for 30 s, annealing at 56°C for 45 s and extension at

72°C for 60 s, with a final extension at 72°C for 5 min. The PCR products were purified and sequenced by Shanghai Majorbio Bio-pharm Technology Co., Ltd. (Shanghai, China). The phylogenetic tree was constructed by the neighbor-joining method using the software MEGA X. The obtained sequencing data were analyzed using NCBI-BLAST, and the 16S rDNA sequence is shown as SEQ ID NO.1 (Fig. S2). Based on the sequence alignment results, strain DQ-4 was identified as *Bacillus* sp.

Text S3. Scanning electron microscope.

The screened strain DQ-4 was inoculated into LB medium and cultured to the logarithmic phase. The resulting culture was streaked onto LB agar plates and incubated at 30°C for 48 h. Single colonies were then isolated (Fig. S3a, b). Strain DQ-4 formed circular, well-defined colonies with a viscous surface and a dark-yellow coloration. Strains of logarithmic growth stage were centrifuged and washed three times with sterile water. Cells were fixed by glutaraldehyde and then dehydrated with 20%, 30%, 50%, 70%, 90%, and 100% ethanol. The dried cells were glued onto scanning electron microscopy (SEM; FEI-Verios 460 L, USA) stubs and gold-coated, and then SEM images were obtained at an accelerating voltage of 3.0 kV. Under the scanning electron microscope strain, as shown in Fig. S3c, d, it is short rod-shaped, approximately 2μm in size.

Text S4. Optimization of fermentation conditions for strain DQ-4.

Culture of strain DQ-4: After 24 h of enrichment culture, inoculate 5% of the bacteria into each of the following selective media. Incubate at an initial pH of 7.5, 35°C, and 150 rpm in a constant-temperature shaking incubator for 3 days.

(1) Carbon source selection

Weigh 20 g/L of sucrose, n-hexadecane, glycerol, glucose, olive oil, sodium citrate, and liquid paraffin inorganic salt medium separately. Determine the OD600, surface tension, and emulsification index of the fermentation broth, then extract the surfactant produced after fermentation. Select suitable carbon sources based on the surface tension of the solution and the yield of surfactants after fermentation.

Fig. S4 illustrates the effects of different carbon sources on bacterial growth, fermentation broth surface tension, and DQ-4 surfactant production. When n-hexadecane, olive oil, and liquid paraffin were used as carbon sources, bacterial growth was slow and substrate conversion rates were low. However, the surface tension of solutions using n-hexadecane and olive oil as carbon sources was lower because these substrates could not be fully utilized, leaving residues in the solution that affected surface tension measurements. Using glucose as the carbon source resulted in better bacterial growth, higher surfactant yield, and the lowest surface tension. Glucose, being a simple organic compound, exhibits high microbial utilization efficiency and readily dissolves in water, avoiding interference with subsequent product extraction. Therefore, glucose was selected as the carbon source for fermentation in all subsequent experiments.

(2) Temperature selection

Prepare an inorganic salt medium using glucose (20 g/L) as the carbon source. Adjust the initial pH of the inorganic salt medium to 7.5. Incubate at fermentation temperatures of 15, 25, 35, 45, and 55°C for 3 days. Measure the OD_{600}, surface tension, and emulsification index of the fermentation broth. Extract the surfactant produced after fermentation. Select the optimal temperature based on the surface tension of the solution and the yield of surfactant produced after fermentation.

Fig. S5 indicates that the optimal fermentation temperature for this biosurfactant is 15°C. At temperatures below this threshold, microbial growth slows and substrate conversion rates decline. Conversely, at 15°C, microbial growth is robust and metabolic activity is optimal. Temperatures above 15°C inhibit microbial growth and cause a sharp decrease in biosurfactant synthesis. Therefore, 15°C was selected as the cultivation temperature for this strain.

(3) pH selection

Prepare an inorganic salt medium with glucose (20 g/L) as the carbon source. Ferment at 15°C, adjusting the initial pH of the inorganic salt medium to 4, 6, 7, 8, and 10, respectively, and incubate for 3 days. Measure the OD_{600}, surface tension, and emulsification index of the fermentation broth. Extract the surfactant produced after fermentation. Select the optimal pH based on the surface tension of the solution and the yield of surfactant after fermentation.

As shown in Fig. S6, when the initial pH is around 7, the fermentation broth exhibits lower surface tension and yields the highest production. However, when the initial pH falls below 7 or rises above 7, the substrate conversion rate decreases sharply. Therefore, an initial pH of 7.0 was selected for the fermentation medium.

(4) Selection of cultivation period

Prepare an inorganic salt medium using glucose (20 g/L) as the carbon source. Adjust the initial pH of the inorganic salt medium to 7. Cultivate at a fermentation temperature of 15°C for 1, 3, 5, and 7 days, respectively. Measure the OD_{600}, surface tension, and emulsification index of the fermentation broth. Extract the surfactant produced after fermentation. Select the optimal incubation duration based on the surface tension of the solution and the yield of surfactant produced after fermentation.

As shown in Fig. S7, at 1 day of cultivation, the strain has just reached the growth plateau phase. The carbon source remains largely unconsumed, and substrate conversion is ongoing. resulting in a relatively low conversion rate. By the third day of cultivation, substrate conversion rates increase significantly, the surface tension of the fermentation broth decreases, and yield reaches its peak. However, by the seventh day, as the carbon source is largely depleted and available nutrients for the strain are exhausted, the bacterial concentration begins to decline. The strain then starts consuming its own metabolites. Therefore, the optimal fermentation time is determined to be 3 days.

After optimization, the optimal carbon source was glucose (20 g/L), the optimal cultivation temperature was 15°C, the optimal initial pH was 7, and the optimal cultivation time was 3 days. Under these optimized conditions, the final surface tension

reached 34.7 mN/m, OD_{600} was 1.724, the emulsification index was 72.3%, and the yield was 14.263 g/L.

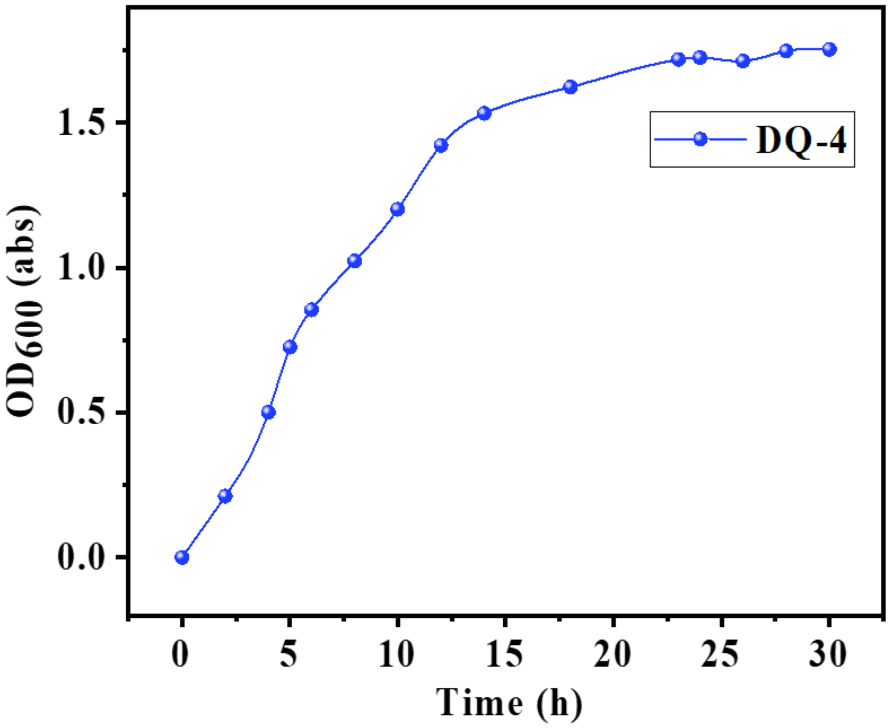

Fig. S1. The growth curve of strain DQ-4 in the enrichment medium

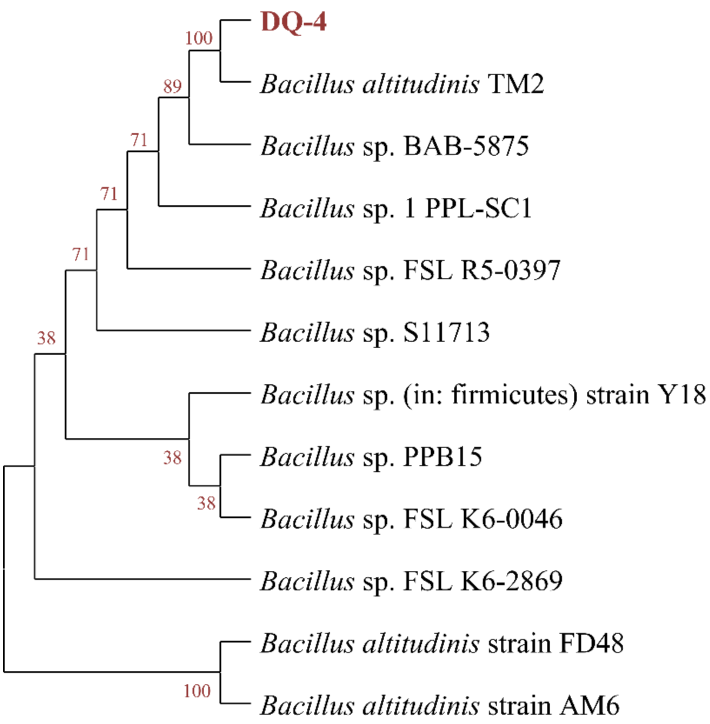

Fig. S2. The phylogenetic tree of strain DQ-4

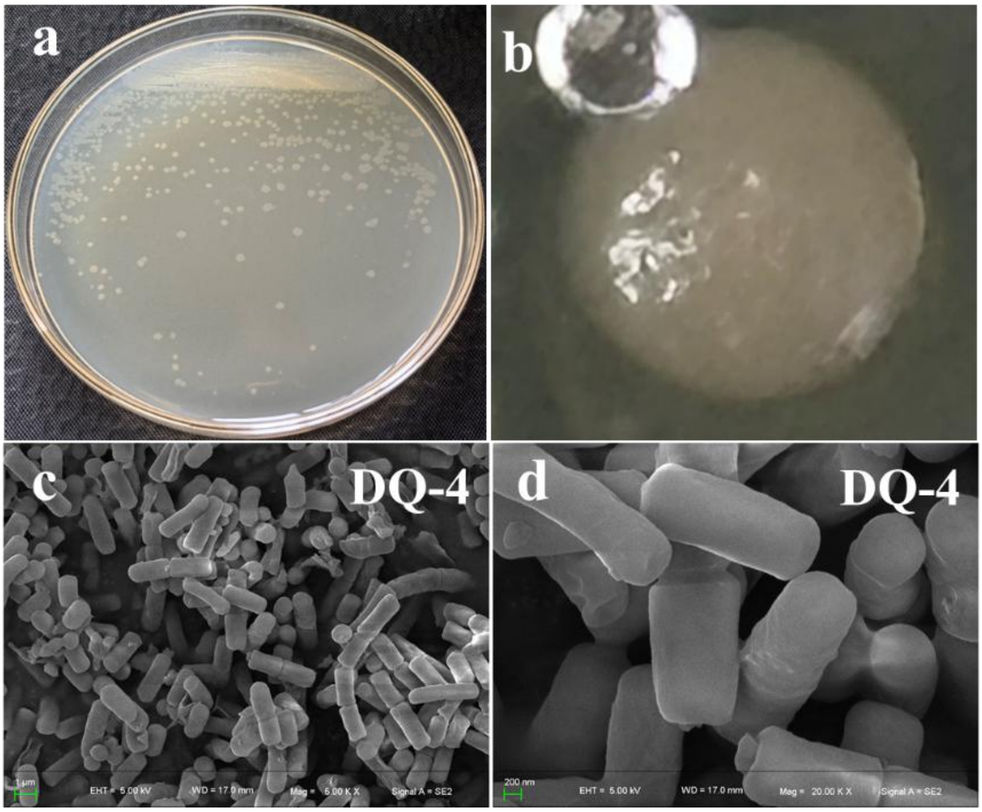

Fig. S3. (a) Plate image of strain DQ-4; (b) Colony image of strain DQ-4; (c, d) SEM image of strain DQ-4

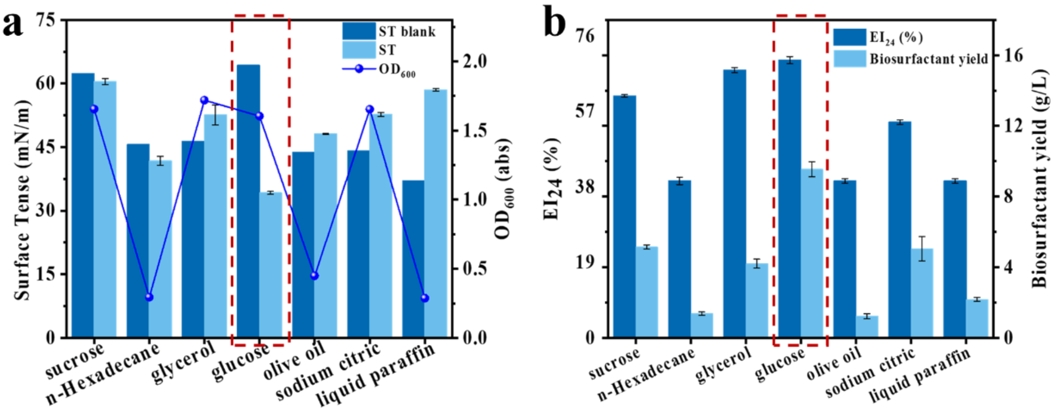

Fig. S4. The growth and metabolic conditions of DQ-4 under different carbon sources

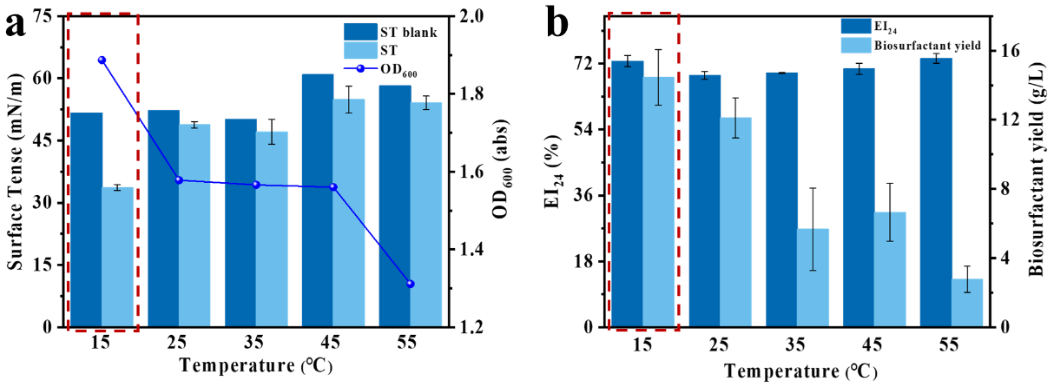

Fig. S5. The growth and metabolic conditions of DQ-4 at different temperatures

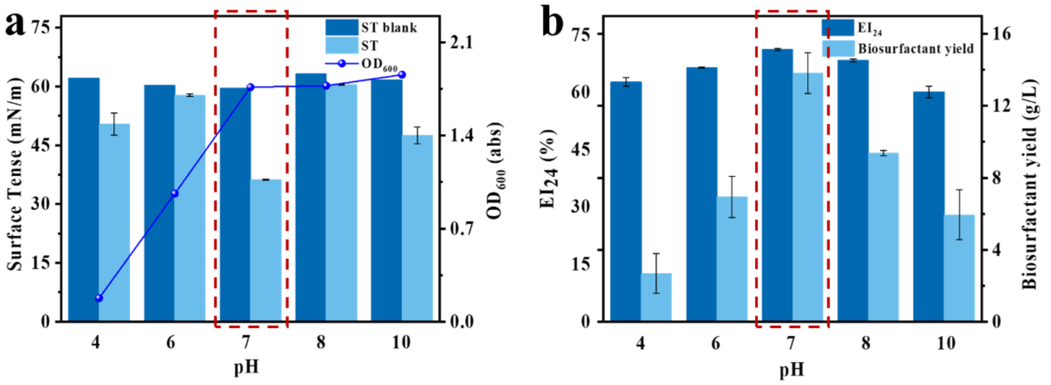

Fig. S6. The growth and metabolic conditions of DQ-4 under different pH values

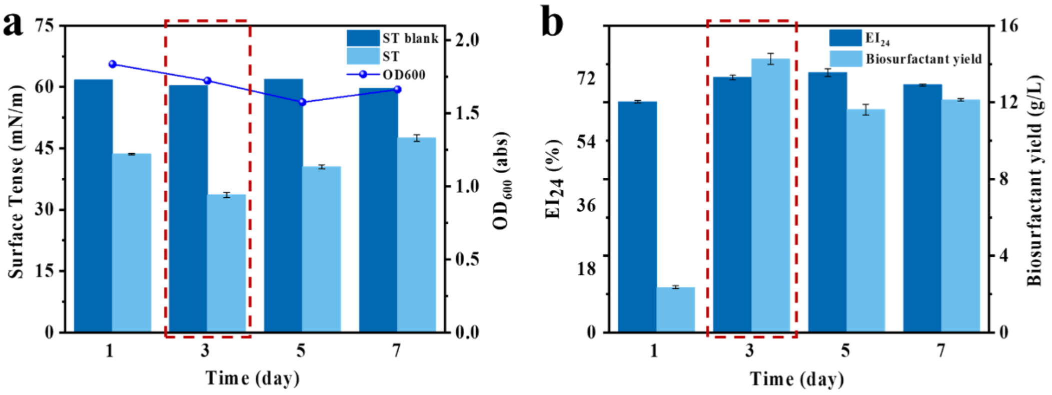

Fig. S7. The growth and metabolic conditions of DQ-4 at different culture days
